# The Chinese version of the American shoulder and elbow surgeons standardized shoulder assessment form questionnaire, patient self-report section: a cross-cultural adaptation and validation study

**DOI:** 10.1186/s12891-021-04255-z

**Published:** 2021-04-24

**Authors:** Tung-Hee Albert Tie, Chih-Kai Hong, Illich Chua, Fa-Chuan Kuan, Wei-Ren Su, Kai-Lan Hsu

**Affiliations:** 1 National Cheng Kung University Hospital, College of Medicine, National Cheng Kung University, No.138, Sheng-Li Road, North Dist., Tainan City, 70428 Taiwan; 2 Department of Orthopaedic Surgery, National Cheng Kung University Hospital, College of Medicine, National Cheng Kung University, No.138, Sheng-Li Road, North Dist., Tainan City, 70428 Taiwan; 3 Department of Pharmacy, College of Medicine, National Cheng Kung University, No. 1, University Road, East Dist., Tainan City, 70101 Taiwan; 4 Department of Biomedical Engineering, National Cheng Kung University, No.138, Sheng-Li Road, North Dist., Tainan City, 70428 Taiwan; 5 Skeleton Materials and Bio-compatibility Core Lab, Research Center of Clinical Medicine, National Cheng Kung University Hospital, No.138, Sheng-Li Road, North Dist., Tainan City, 70428 Taiwan

**Keywords:** ASES score, Cross-cultural adaptation, Validation, Patient self-reported questionnaire

## Abstract

**Background:**

The patient self-report section of the American Shoulder and Elbow Surgeons Standardized Shoulder Assessment Form (ASESp) is one of the most validated and reliable assessment tools. This study aimed to establish a validated Chinese version of ASESp (ASESp-CH).

**Methods:**

A clinical prospective study was performed (ClinicalTrials.gov Identifier: NCT04755049; registered on 2021/02/11). Following the guidelines of forward-backward translation and cross-cultural adaptation, a Chinese version of ASESp was established. Patients older than 18 years with shoulder disorders were included. Patients who could not complete test-retest questionnaires within the interval of 7–30 days and patients who received interventions were excluded. Intraclass correlation (ICC) was calculated for test- retest reliability, whereas internal consistency was determined by Cronbach value. Construct validity was evaluated by comparing the corresponding domains between the ASESp-CH and a validated Chinese version of 36-Item Short Form Health Survey (SF-36).

**Results:**

A total of 86 patients were included with a mean test-retest interval of 12 ± 5.4 days. Test-retest reliability was excellent with an ICC of 0.94. Good internal consistency was found, with a Cronbach alpha of 0.86. Construct validity of the ASESp-CH questionnaire was good. The major domains of the ASESp-CH were significantly correlated with the respective domains in the SF-36 (*p* <  0.01), except for the domain of stability of ASESp-CH.

**Conclusions:**

The Chinese version of ASESp questionnaire is a highly validated and reliable tool for shoulder disorder assessment.

**Supplementary Information:**

The online version contains supplementary material available at 10.1186/s12891-021-04255-z.

## Background

Shoulder pain is one of the most common musculoskeletal disorders in the human population [1–3]. A shoulder pathology may lead to pain, disability, and immobility which eventually affect one’s activity of daily living (ADL) and quality of life (QOL) resulting in a socio-economic burden [2, 4, 5]. Similarly, elbow pain due to instability or consequences of traumatic events compromised one’s QOL as well [6]. To reach a better prognosis in approaching shoulder and elbow disorders, functional improvement is an essential outcome. Apart from clinician-reported outcome instruments, patient-reported outcome measures (PROMs) are recognized to be essential and increasingly used to quantify a patient’s perceptions of functional ability in recent decades [4, 7]. There are several PROMs available to evaluate a patient’s condition, the patient self-evaluation section of the American Shoulder and Elbow Surgeons Standardized Shoulder Assessment Form Questionnaire (ASESp), one of the most widely used PROMS at the present time, is a highly validated and reliable PROM compare to the similar items [4, 5]. It is easy and quick to use this validated questionnaire for evaluating shoulder problems [8, 9].

Due to diversity in the culture and natives’ languages in different regions, a cross-cultural adaptation of ASESp shall be established. Currently, the ASESp questionnaire has been translated and validated into different languages to accommodate the clinical practices and research-needs including Dutch, Italian, Finnish, Spanish, German, Portuguese, Turkish, Arabic and Argentina [4, 8, 10–15].

The language is an indirect indicator of culture, an incorrect interpretation due to a non-validated translated questionnaire could lead to differences in functional score obtained and thus, low credibility [4]. Apart from minimizing the language barrier, having a validated scale in the local language is beneficial to clinical practice and future research in Chinese speaking countries. Currently, several Chinese versions of ASESp were attainable on the Internet; however, a unified and validated version has yet to be found. The purpose of this study was to conduct the translation and establish a cross-cultural adapted and validated Chinese version of the ASESp score (ASESp-CH).

## Methods

The present study was officially authorized by the American Shoulder and Elbow Surgeons Society, the original developer of ASESp. Informed consents were obtained from all participants prior to the study.

### ASES questionnaires

ASES composed of 3 sections: demographic information, patient self-evaluation (ASESp), and physician assessment [8]. A clinician is responsible to provide his or her expertise to evaluate the range of motion, strength, instability and other shoulder pathology signs; however, a score index can only be derived from the ASESp section for a thorough assessment [14].

The ASESp consists of 18 questions from 3 sections: pain, instability, and activities of daily living (ADL). Among the 18 questions, 11 self-report items representing functional (ADL) dimension (10 items) and pain dimension (1 item) are derived into a 0–50 sub-score for each dimension [14]. The ADL section was scored in a 4-points- graded ordinal scale, ranging from 0 (unable to do) to 3 (not difficult) and cumulative scores were collected. The pain section was derived from the 10-points-graded visual analog scale (VAS) ranging from 0 (no pain) to 10 (maximum pain) [7]. The overall shoulder score index was calculated with the formula below, ranging from 0 (most disability) to 100 (least disability) [8].

$$ \mathrm{Shoulder}\ \mathrm{score}\ \mathrm{index}=\left[\left(10-\mathrm{VAS}\ \mathrm{pain}\ \mathrm{score}\right)\ \mathrm{x}\ 5\right]+\kern0.5em \left[\left(5/3\right)\ \mathrm{x}\ \mathrm{cumulative}\ \mathrm{ADL}\ \mathrm{score}\right] $$

### Translation and linguistic validation

A 5 steps protocol of forward-backward translation was derived based on the Beaton’s method published in the Guidelines for the Process of Cross-Cultural Adaptation of Self-Report Measures [16]. The protocol included translation, synthesis, back-translation, expert committee review and pretesting [16–20].

#### Step 1: forward translation from English to Chinese

A forward translation was done separately by two native Chinese native speakers, one acting as an informed translator (senior orthopedic resident) and one acting as an un-informed translator (medical student).

#### Step 2: cross-culture adaptation

Several cross-cultural dissimilarities were found in the first translated version. First, in question 4, the example of pain medications given in original ASES, namely aspirin, Tylenol, Advil, Codeine, were replaced with aspirin and acetaminophen together with the respective Chinese brand name. Similarly, the narcotic medication given in question 5 was also replaced with tramadol, Ultracet and morphine together with the respective Chinese brand name. These modifications were done based on the most common, most familiar and typical prescription in Taiwan. Secondly, we clearly define the description of “Manage Toileting” in question 4 of the ADL section as a “butt-wiping” situation. Third, the “10 lbs” in question 7 of the ADL section was translated and remarked as “5 kg” as kilogram, which was a typical measurement unit in Taiwan.

#### Step 3: backward translation from Chinese to English

A backward translation was done by an English native speaker who was not familiar with the orthopedics field after a Chinese consensus version was completed.

#### Step 4: revision by expert committees

Both forward and backward versions were then revised and reviewed by an expert committee, which composed of five senior orthopaedic surgeons, including the chief of department of orthopaedic surgery. Both versions revealed no marked disparity or language difficulties. Thus, the primary Chinese ASES questionnaire (ASESp-CH) was formed.

#### Step 5: pre-test of ASESp-CH questionnaires

**Fig. 1:**
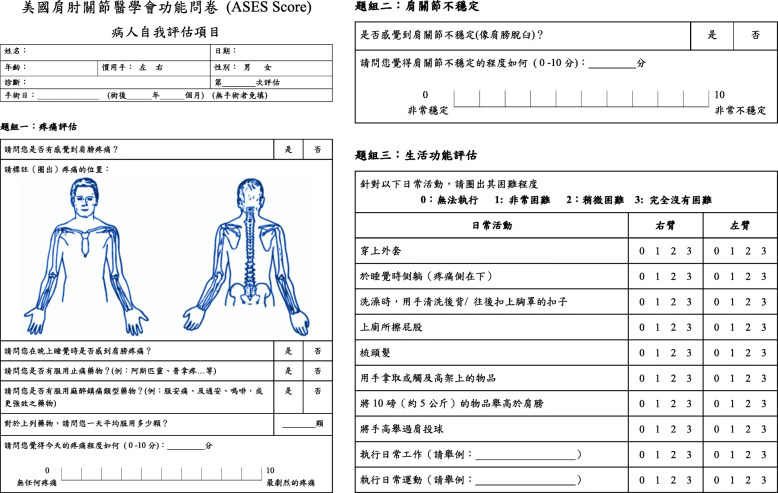
Chinese version of the American Shoulder and Elbow Surgeons Standardized Shoulder Assessment Form (ASESp-CH)

The ASESp-CH questionnaire was given to 20 patients to disclose any problem in understanding and approaching the questionnaire. There were no obstacles reported. Hence, a final ASESp-CH questionnaire was established (Fig. 1).

### Study population

The study was conducted by the Department of Orthopedic Surgery in National Cheng Kung University Hospital (NCKUH) in Tainan, Taiwan, and was approved by the Institutional Review Board of National Cheng Kung University Hospital. Patients with shoulder disorder were recruited from the out-patient department of NCKUH and the public population. All patients were required to complete the test-retest ASESp-CH questionnaires twice at the interval of 7 days to 30 days before getting any intervention. A questionnaire of SF-36 was also required to complete during the retest session of ASESp-CH. A thorough explanation and informed consent were given.

Inclusion criteria were as follow: [1] patients’ age ≥ 18 years, [2] patients with clear insights, [3] patients with any shoulder disorders, [4] patients who are able to speak and write in Chinese, and [5] patients who completed the questionnaires twice at an interval of 7 days to 30 days. Patients with one of the following conditions were excluded: [1] the patient could not complete all of ASESp-CH and SF-36 questionnaires, [2] the test-retest interval was less than 7 days or more than 30 days, and [3] the patient received interventional procedures, such as shoulder injections or surgery, during the test-retest interval.

### Reliability

Reliability was considered as the degree of replicable, is the extent to which the results can be reproduced when the research is repeated under the same conditions. In this case, refers to the degree of the results of ASESp-CH can be replicated in a test-retest manner and among related items on the ASESp-CH (internal consistency) [8]. Reliability can be expressed ranging from 0 to 1, indicates no reliability and absolute reliability respectively.

Internal consistency is calculated by using the Cronbach alpha, a widely recognized analysis tool to evaluate one’s reliability [21]. This method has been used in previous ASESp validation studies [4, 8, 10–15]. A cut-off value of 0.7 represents a sufficient correlation between the items of a questionnaire. Values between 0.7 to 0.79, 0.80 to 0.89 and above 0.90 imply fair, good and excellent respectively. Yet, a Cronbach alpha greater than 0.90 may indicate a highly homogenous situation and thus redundant [8].

While considering the test-retest reliability, it was assumed that 2 separate measurements in a suitable interval should be similar if no change occurs and the time-bias can be reduced to the minimum level. A short interval may cause memory bias, while a longer interval may encounter actual changes in patient health status [8]. Thus, a time interval between 7 to 30 days was considered relevant according to the guidelines of cross-cultural adaptation. To calculate the test-retest reliability, the intraclass correlation coefficient (ICC) was used [8, 22]. An ICC of 0 indicated no agreement, whereas an ICC of 1 indicated absolute agreement. Generally, an ICC greater than 0.60 and 0.74 were considered good and excellent, respectively [22].

### Validity

To achieve the validation of ASESp-CH, the results collected from 3 domains of pain, instability, and ADL are compare with the corresponding 8 domains of 36-Item Short Form Survey (SF-36) [23]. As a widely accepted and validated health status measure, SF-36 also has been used as a parameter for the various translated versions of ASESp in the previous studies [4, 8, 10–14]. A translated and validated Taiwan version of SF-36 was eligible for the validation process [24, 25].

Pearson correlation coefficient was used to evaluate the construct validity between the 3 domains of ASESp-CH and the corresponding domains in SF-36. Statistical analysis was performed using SPSS 20.0 (IBM, Armonk, NY, USA) and Excel 2010 (Microsoft, Redmond, WA, USA). A *p* <  0.05 was considered statistically significant.

## Results

In this study, a total of 112 patients with shoulder disorders were asked to participate from March 1st, 2020 to September 30th, 2020. Sixteen patients who refused to participate and 10 patients who did not complete the required questionnaires were excluded. Finally, 86 of the patients were included and the patients’ demographic data were shown in Table 1.

**Table 1:** Patient characteristics (*n* = 86)

	No. (%)
Sex	
Female	30 (34.9)
Age, year, mean (SD)	39.2 (± 17.6)
Test-retest interval, days, mean (SD)	12.4 (± 5.3)
Affected side	
Right shoulder	54 (62.8)
Left shoulder	28 (32.6)
Bilateral shoulder	4 (4.7)
Diagnosis	
Rotator cuff tear	25 (29.1)
Frozen shoulder	5 (5.8)
Labrum lesions	14 (16.3)
Shoulder osteoarthritis	1 (1.2)
Shoulder muscle sprain	37 (43.0)
AC joint lesion	2 (2.3)
Calcified tendinitis	1 (1.2)
Unknown	1 (1.2)

*SD* Standard deviation, *AC* Acromioclavicular

### Internal consistency and test-retest reliability

The Internal Consistency of our study was good, the Cronbach alpha value being 0.86. The mean interval of the test-retest process was 12 days (S.D 5.4 days). The results of test-retest reliability showed excellent findings, with an ICC of 0.94 (95% confidence interval 0.90,0.96; *p* <  0.01) for the total ASESp-CH score. The ICCs for each domain of ASESp-CH were all greater than 0.80 (Table 2).

**Table 2:** Test-retest reliability

ASESq domains	ICC (95% CI)	***P*** value
Pain	0.83 (0.75–0.89)	< 0.01
Stability	0.85 (0.78–0.90)	< 0.01
Daily activities	0.96 (0.93–0.97)	< 0.01
ASESq total scores	0.94 (0.90–0.96)	< 0.01

### Construct validity

The major domains of the ASESp-CH were significant correlated with the respective domains in the SF-36 (*p* <  0.01), except for the stability domain of ASESp-CH (Table 3). Moreover, the ASESp-CH total score was especially highly correlated (correlation > 0.7) to the Physical Function, Role Limitation-Physical and Bodily Pain domain in the SF-36 (*p* <  0.001).

**Table 3:** Correlation between ASESq domains and the domains of the SF-36 questionnaire

SF-36 domains	Physical function	General health	Vitality	Mental health	Role limitations-physical	Role limitations-emotional	Social functioning	Bodily pain
ASESq domains								
Pain								
Correlation	−0.568^a^	− 0.385^a^	− 0.446^a^	−0.367^a^	− 0.674^a^	−0.366^a^	− 0.323^a^	−0.701^a^
Significance (2-tailed)	< 0.001	< 0.001	< 0.001	< 0.001	< 0.001	< 0.001	0.002	< 0.001
Stability								
Correlation	0.038	−0.145	− 0.195	−0.150	0.007	0.041	0.062	−0.027
Significance (2-tailed)	0.725	0.183	0.073	0.169	0.951	0.705	0.572	0.807
Activity								
Correlation	0.802^a^	0.386^a^	0.418^a^	0.412^a^	0.825^a^	0.547^a^	0.485^a^	0.784^a^
Significance (2-tailed)	< 0.001	< 0.001	< 0.001	< 0.001	< 0.001	< 0.001	< 0.001	< 0.001
Total								
Correlation	0.760^a^	0.424^a^	0.478^a^	0.431^a^	0.830^a^	0.507	0.447^a^	0.821^a^
Significance (2-tailed)	< 0.001	< 0.001	< 0.001	< 0.001	< 0.001	< 0.001	< 0.001	< 0.001

*ASESq* American Shoulder and Elbow Surgeons Standardized Shoulder Assessment Form, *SF-36* 36-Item Short Form Health Survey

^a^Correlation was significant at the alpha < 0.05 level (2-tailed)

## Discussions

As the patient-reported outcome measures (PROMs) are emphasized in clinical practice, the ASESp, a highly validated and reliable PROM, becomes a popular choice for measuring shoulder function [4, 5, 8]. Due to language and cultural diversity, ASESp had been translated into different languages across the world [4, 8, 10–15]. However, to our knowledge, a validated Chinese ASESp has not been proposed. The aim of the present study was to establish a Chinese version of ASESp, as known as the Chinese ASES questionnaire (ASESp-CH), to assess its reliability and validity. Our results showed that the proposed ASESp-CH was a validated and reliable tool for shoulder function assessment in this study.

During the translation processes, cross-cultural adaptions were necessary. Undoubtedly, some cross- cultural dissimilarities were found in the present study. Similar to the previous study [8], the major amendments were made due to cultural differences, such as the examples of pain control medicine, unit of weight, etc. Additionally, we also clearly define the description of “Manage Toileting” in question 4 of the ADL section as a “butt-wiping” situation because of the cultural and language difference. After adequate adaption, no serious difficulties in realizing the statements were reported during pretesting.

Our study achieved a great result of internal consistency with a Cronbach alpha of 0.86. Cronbach Alpha values between 0.80 and 0.89 were regarded as good internal consistency [26]. Reviewing the previous adaptation studies [4, 8, 10, 12, 13], most of the Cronbach Alpha values were in the range of 0.80–0.90. The Cronbach alpha value in our study was similar to the aforementioned studies [4, 8, 10, 12, 13].

Regarding about test-retest reliability, an excellent ICC of 0.94 was found for the total ASESp-CH score. Compared with the prior studies [4, 8, 10, 12, 13], test-retest reliability in the present study was relatively great, even the mean test-retest interval reached 12 days. The finding was impressive, as the previous studies [8, 11] suggested that a longer test-retest interval could reduce memory bias yet result in lower ICC. Therefore, the ASESp-CH could be considered a well-translated and well-constructed questionnaire that could be fully-understand without too many interpretation errors.

The present study also evaluated the construct validity for the ASESp-CH. We compared the domains of the ASESp-CH with the domains of the validated and translated Chinese instruments Short-Form 36 questionnaire (SF-36) [24, 25]. A good construct validity was found for the domains of ASESp-CH to the corresponding domains in SF-36, except for the stability domain. It is exciting to find that the ASESp-CH was significantly correlated to all of the domains of SF-36, although the previous studies of other language versions were only correlated to some SF-36 domains [8, 10]. Furthermore, the ASESp-CH was highly correlated to physical functioning, role limitation-physical and bodily pain domains of SF-36, suggesting the ASESp-CH to be an appropriate tool for clinical evaluation.

Unfortunately, the domain of stability was not significant correlated to SF-36 in our study. Although the previous study [8] reported significant correlation between the stability and some SF-36 domains, only modestly correlation was identified. The possible reason for the above findings was that the clinical definition of instability could not be fully understood by the patient without instability. From the clinical observation during the study, some patients have questions regarding the definition of instability, even though an example, sensation of shoulder dislocation, has been given in the questionaire. To minimized the misunderstanding in this section, we suggested that adequate explanation of shoulder instability should be given to the patients before filling out the questionaires.

## Limitations

The present study had some limitations. First, there was no final consensus on the way of cross-cultural adaptation and validation. The present study selected the most respected guidelines, which were also used by previous ASESp adaptation and validation studies. Second, a large proportion of senior elders with age above 75 were not able to speak, read, or even write in Chinese. The patients were unfortunately excluded in this study, which possibly leading to a selection bias and the compromised reliability in elderly population. Third, although the sample size of our study considered satisfied, however, the ratio of gender was not well-balanced as the aforementioned studies. Lastly, Chinese is a commonly used language in several countries and regions, including Taiwan, China, Hong Kong, Macau, South-east Asia etc. Care should be taken while using the translated questionnaire in our study because the cultural difference was noticeable and some brand names of medicine were not identical among different regions. Therefore, minor revision of the current questionnaire is suggested before application in different regions.

## Conclusions

The Chinese version of ASES questionnaire is a highly validated and reliable tool for shoulder disorder assessment.

## Supplementary Information

**Additional file 1.** ASESp Questionnaire.

## Data Availability

The datasets used and analyzed during the current study are available from the corresponding author on reasonable request.

## Abbreviations

ASESp: American Shoulder and Elbow Surgeons Standardized Shoulder Assessment Form
ASESp-CH: Chinese version of ASESp
ICC: Intraclass correlation
SF-36: 36-Item Short Form Health Survey
ADL: Activities of daily living
QOL: Quality of life
VAS: Visual analog scale
PROM: Patient-reported outcome measures
