# Supplementary material for: The Chinese version of the American shoulder and elbow surgeons standardized shoulder assessment form questionnaire, patient self-report section: a cross-cultural adaptation and validation study
Source: BMC Musculoskelet Disord. 2021 Apr 24;22:382. doi: 10.1186/s12891-021-04255-z (PMC8070278; doi:10.1186/s12891-021-04255-z)
Supplement: Supplementary file 1 — Additional file 1. ASESp Questionnaire. [file 12891_2021_4255_MOESM1_ESM.docx]

ASESp Questionnaire

| PATIENT SELF-EVALUATION | | |
| --- | --- | --- |
| Are you having pain in your shoulder? (circle correct answer) | Yes | No |
| Mark where your pain is:  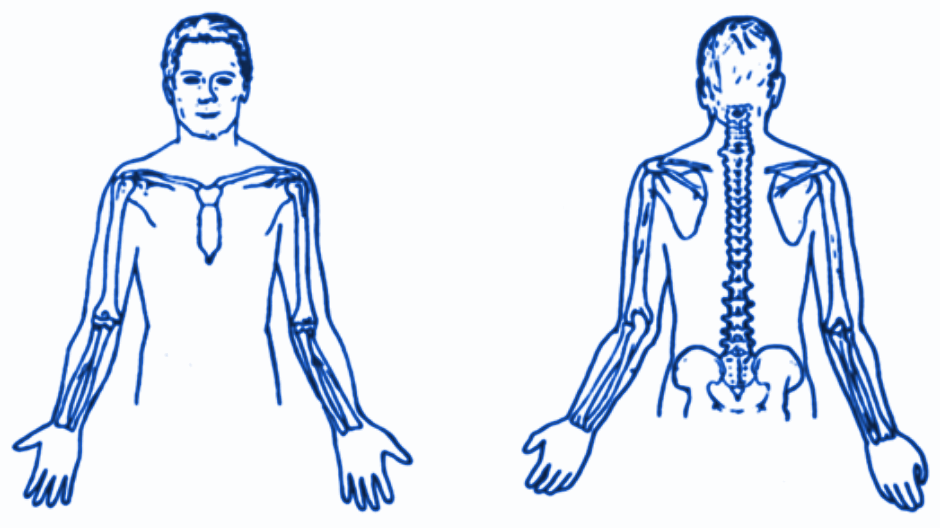 | | |
| Do you have pain in your shoulder at night? | Yes | No |
| Do you take pain medication (aspirin, Advil, Tylenol etc.)? | Yes | No |
| Do you take narcotic pain medication (codeine or stronger)? | Yes | No |
| How many pills do you take each day (average)? | ______ pills | |
| How bad is your pain today (mark line)?  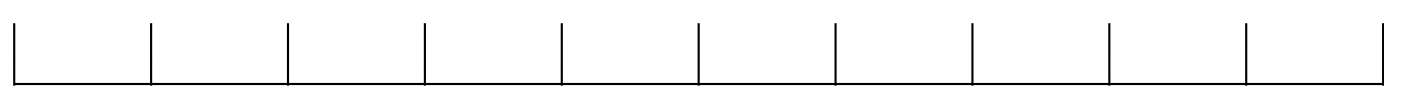  0 10  No pain at all Pain as bad as it can be | | |

| How unstable is your shoulder (mark line)?  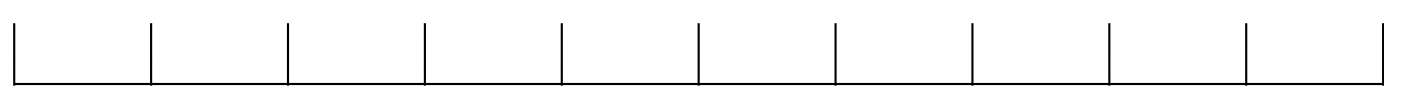  0 10  Very Stable Very Unstable |
| --- |

| Circle the number in the box that indicates your ability to do the following activities:  0 = **Unstable** to do; 1 = **Very** difficult to do; 2 = **Somewhat** difficult; 3 = **Not** difficult | | | |
| --- | --- | --- | --- |
| Activity | | Right Arm | Left Arm |
| 1. Put on a coat | | 0 1 2 3 | 0 1 2 3 |
| 1. Sleep on your painful or affected side | | 0 1 2 3 | 0 1 2 3 |
| 1. Wash back/ do up bra in back | | 0 1 2 3 | 0 1 2 3 |
| 1. Manage toileting | | 0 1 2 3 | 0 1 2 3 |
| 1. Comb hair | | 0 1 2 3 | 0 1 2 3 |
| 1. Reach a high shelg | | 0 1 2 3 | 0 1 2 3 |
| 1. Lift 10 lbs. above shoulder | | 0 1 2 3 | 0 1 2 3 |
| 1. Throw a ball overhead | | 0 1 2 3 | 0 1 2 3 |
| 1. Do usual work- List: |  | 0 1 2 3 | 0 1 2 3 |
| 1. Do usual sport- List: |  | 0 1 2 3 | 0 1 2 3 |
